# 

**Published:** 1892-04-02

**Authors:** 


### CONTENTS.

Phagocytosis - - - - - - - - - - - 1

Passing Topics.—The Long-suffering Middle-class - - - 2

The Doctor's Armchair.—Oxygen at Oxford - - - - 3

A Model Prison - - - - - - - - - - 4

Medical History from the Earliest Times.—By E. T. Withington, M.A., M.B., Oxon.—II. Medicine in Prehistoric Times - - - - - - - - - - - - - 5

The Story of the Insane from Year to Year - - - 6

Everybody's Page - - - - - - - - - 7

Notes and News, 8; Scraps and Gleanings - - - 9

Annotations.—Our New Departure—Some Good Women—The "Year Book of Science" - - - - - - - - - 10

The Practitioner's Mirror and Retrospect—

Medicine.—Intubation in Laryngeal Tuberculosis - - - 11

Surgery.—The Symptoms of Surgical Affections of the Kidneys 12

New Drugs, Appliances, and Things Medical.—Improvement in Syphons - - - - - - - - - - 12

The Institutional Workshop—The Chronic Indigence of our Hospitals, with some Suggestions for its Remedy. By Burford Rawlings. I.—A Bird's Eye View - - - 13

Hospital Construction—The Mary Hitchcock Memorial Hospital, Hanover, N.H., U.S.A. - - - - - - - 15

The Student of Medicine.—Appointments—Vacancies—Notes from the Schools—Pass Lists—Athletics.

### EXTRA SUPPLEMENT.—THE NURSING MIRROR.

En Passant.—Burton Institution—The Private Nurse—Nurses' Beneficial Association—Sunderland Institute—Birmingham Institution—St. Mary's Home, Southampton—Little Children—Bicester Nursing Home—The Registration of Nurses - - i

The Nursing of the Insane.—By a Medical Officer - - ii

Appointments - - - - - - - - - - ii

Death in Our Ranks - - - - - - - - - ii

Lady Doctors in India - - - - - - - - - iii

For Reading to the Sick.—Day by Day - - - - - - iii

A Private Asylum - - - - - - - - - iv

Everybody's Opinion—Queen Victoria's Jubilee Institute - iv

Examination Questions - - - - - - - - iv

Off Duty.—"Mrs. Pharisee" - - - - - - - v

Where to Go - - - - - - - - - - v

Notes and Queries - - - - - - - - - v

Talks about Food. VI.—Drink - - - - - - vi

